# Kupffer cell-derived TNF-α promotes hepatocytes to produce CXCL1 and mobilize neutrophils in response to necrotic cells

**DOI:** 10.1038/s41419-018-0377-4

**Published:** 2018-02-23

**Authors:** Li Su, Na Li, Hua Tang, Ziyang Lou, Xiaodan Chong, Chenxi Zhang, Jiacan Su, Xin Dong

**Affiliations:** 1 School of Pharmacy, Second Military Medical University, Shanghai, China; 2 Cancer Institute, Institute of Translational Medicine, Second Military Medical University, Shanghai, China; 3 Department of Orthopedics Trauma, Changhai Hospital of Shanghai, Second Military Medical University, Shanghai, China

## Abstract

The damage-associated molecular pattern molecules (DAMPs) released by necrotic cells can trigger inflammatory response, which will facilitate the clearance of these dead cells. Neutrophil mobilization is a very important step for the dead cell clearance, however the detailed mechanisms for DAMPs induce neutrophil mobilization remains largely elusive. In this study, by using a necrotic cell-induced neutrophil mobilization mice model, we found that both neutrophil number and percentage rapidly (as early as 30 min) increased with necrotic cells but not live cell treatment. CXCL1 was rapidly increased in the serum and was responsible for the neutrophil mobilization when treated with necrotic cells. We further demonstrated that the hepatocytes in the liver were the main source of CXCL1 production in response to necrotic cells challenge. However, the hepatocytes did not express CXCL1 when incubating with necrotic cells alone. When Kupffer cells were ablated, the increased CXCL1 levels as well as neutrophil mobilization were abolished with necrotic cells challenge. Moreover, we clarified Kupffer cells-derived TNF-α activates the NF-κB pathway in hepatocytes and promote hepatocytes to express CXCL1. In summary, we showed that the liver is the main source for necrotic cell-induced CXCL1 production and neutrophil mobilization. Kupffer cells in the liver sense DAMPs and release TNF-α to activate the NF-κB pathway in hepatocytes. The interaction between Kupffer cells and hepatocytes is critical for CXCL1 production.

## Introduction

Different from pathogen-associated molecular patterns (PAMPs), which are derived from invading pathogens during microbial infection and provide exogenous alert that the presence of pathogens to immune cells, damage-associated molecular pattern molecules (DAMPs) released by cell death serve as endogenous danger signals that alert the innate immune system and trigger inflammation^1,2^. DAMPs, including HMGB1, mitochondria DNA, heat shock proteins, and purine metabolites, etc, bind to pattern recognition receptors (PRRs) and promote the production of inflammatory mediators such as cytokines and chemokines^1,3^. Necrotic cells, but not apoptotic cells were considered as the most prominent source of DAMPs, the reason may be attribute to the integrity of plasma membrane throughout apoptosis, whereas necrotic cells can release large amount of DAMPs due to the disruption of plasma membranes^4,5^. Innate immune cells, especially neutrophils are mobilized from bone marrow reserve to peritoneal cavity within hours in response to necrotic cell challenge^6^. The mobilization of neutrophils is the critical step for these cells to clear necrotic cell.

In naive mice, around 98% of mature neutrophils reside in the bone marrow, whereas only 2% of total neutrophils are in circulation^7,8^. In normal condition, neutrophils stay in bone marrow because chemokine SDF-1α was constitutively secreted by bone marrow stromal cells^9^. SDF-1α acts as a retention factor for neutrophils in the bone marrow through interacting with its receptor CXCR4^10^. In response to PAMPs during infection, neutrophils are quickly mobilized into blood and fight against invading pathogens by phagocytosis, degranulation, and forming neutrophil extracellular traps (NETs)^11,12^. CXC chemokines, especially CXCL1 is one of the most important specific factor for mobilization of neutrophils from the bone marrow through binding to its receptor CXCR2^8^. Similarly, DAMPs exposure also triggers neutrophil mobilization through PRR TLR9-mediated signaling pathway^13^. CXC chemokines has been described to get involved in neutrophil mobilization in response to DAMPs^6^. However, how CXCL1 expression is regulated and which tissue is the main resource for CXCL1 production response to DAMPs derived from necrotic cells remains unclear.

Here, we treated mice with the necrotic cells and found that neutrophils was mobilized as early as 30 min after challenge. By using this model, we investigated how the “danger signaling” from necrotic cells was sensed and which cells and factors were involved in the CXCL1 production and subsequently neutrophil mobilization.

## Materials and methods

### Animal

Six- to ten-weeks-old male C57BL/6 mice were maintained in a specific pathogen-free facility and were cared for in accordance with animal guidelines. The study was approved by the Institutional Animal Care and Use Committee in Second Military Medical University.

### PBMC isolation

Peripheral blood from mice was collected by cardiac puncture in presence of EDTA. Blood was mixed with PBS (ratio 1:1). The diluted samples were subjected to density gradient separation on Ficoll-Paque (2400 rpm for 30 min). After centrifugation the PBMC layer was collected and washed in PBS.

### Protein extraction

Tissues or PBMCs was homogenized in lysis buffer containing 50 mM Tris pH 7.5, 150 mM NaCl, 1% Triton X-100 and proteinase inhibitors. Supernatants were collected after 12,000 rpm centrifugation for 10 min. Protein concentration was determined by BCA assay.

### Necrotic cells preparation and injection

HEK293 cells were killed by three free-thaw cycles as described previously^14^. A total of 5 × 10^6^ live or necrotic cells were injected into mice by i.v. injections.

### Kupffer cells depletion

To deplete Kupffer cells, mice were given clodronate liposome (FormuMax Scientific, USA) injections (200 μl per mouse) intravenously 24 h prior to necrotic cell injections.

### Primary hepatocytes isolation

Mice were anesthetized and the portal vein was cannulated under aseptic conditions. The liver was subsequently perfused with an EGTA solution and digested with 0.075% collagenase solution. The liver was cut into ~2 mm^3^ piece and shaken for 30 min at 240 rpm in 37 °C incubator and pushed through 70 μm cell strainer. Hepatocytes were collected after centrifugation at 400 rpm for 5 min and seeded on a 6-well plate.

### Blocking antibody and NF-κB inhibitor treatment

CXCL1, IL-6 or TNF-α were blocked by intravenous injection of CXCL1, IL-6, or TNF-α neutralizing antibodies (100 µg per mouse, R&D systems, USA) 15 min before necrotic cell administration. Celastrol (100 µg/kg, Tocris Bioscience, USA) was given to mice 2 h before necrotic cell treatment.

### ELISA

The serum and tissue homogenate levels and of CXCL2, IL-6, IL-1β, IFN-γ, IL-12, and TNF-α were measured by using a Quantikine immunoassay or DuoSet ELISA Development Kits (R&D Systems) according to the manufacturer’s protocol.

### Chemokine levels measurement

Serum levels of a total of 13 chemokines were measured by bead-based immunoassays (Mouse Proinflammatory Chemokine Panel, Biolegend, San Diego, CA, USA) according to the manufacturer’s protocol.

### RNA extraction and real-time PCR

RNA was extracted from mouse tissues or PBMCs by using TRIzol (Life Technologies, USA). Extracted RNA was converted to complementary DNA by using a reverse transcription kit (Applied Biosystems, USA) according to the manufacturer’s instructions. The expression levels of genes were measured with quantitative real-time PCR by using ABI7500 real-time PCR detection system (Applied Biosystems). The primers for mouse genes were shown below:

18S: 5′-AACTTTCGATGGTAGTCGCCGT-3′, 5′-CCTTGGATGTGGTAGCCGTTT-3′

CXCL1: 5′-TCTCCGTTACTTGGGGACAC-3′, 5′-CCACACTCAAGAATGGTCGC-3′

### Immunohistochemistry

Formalin-fixed, paraffin-embedded tissue sections were deparaffinized and rehydrated, followed by antigen retrieval with pH 6 citrate buffer or proteinase K pretreatment. Sections were incubated in 0.3% H_2_O_2_, and followed by another 30 min in 1% BSA. Sections were incubated with primary antibodies overnight at 4 °C. Vectastain Elite ABC Staining Kit and DAB Peroxidase Substrate Kit (Vector Laboratories, USA) were used to visualize the staining according to the manufacturer’s instructions. Primary antibodies used were listed below: anti-myeloperoxidase (MPO) (Biocare Medical, USA), anti-CXCL1 (R&D Systems), anti-F4/80 Ab (Novus Biologicals, USA), NF-κB P65 (Cell Signaling Technology, USA)

### Flow cytometry analysis

PBMCs were stained for Gr-1, CD11b, and CD62L (eBioscience, USA). Stained cells were analyzed on FACSCalibur flow cytometer (BD Biosciences, USA).

### Statistical analysis

The data are shown as mean ± SEM of three independent experiments with 4–10 mice per treatment group. Data from two groups were compared with an unpaired *t*-test. Data from three or more groups were analyzed with one-way ANOVA with Tukey’s multiple comparisons test. Significance was based on *P* values <0.05.

## Results

### Necrotic cells trigger neutrophil mobilization by CXCL1

**Fig.1:**
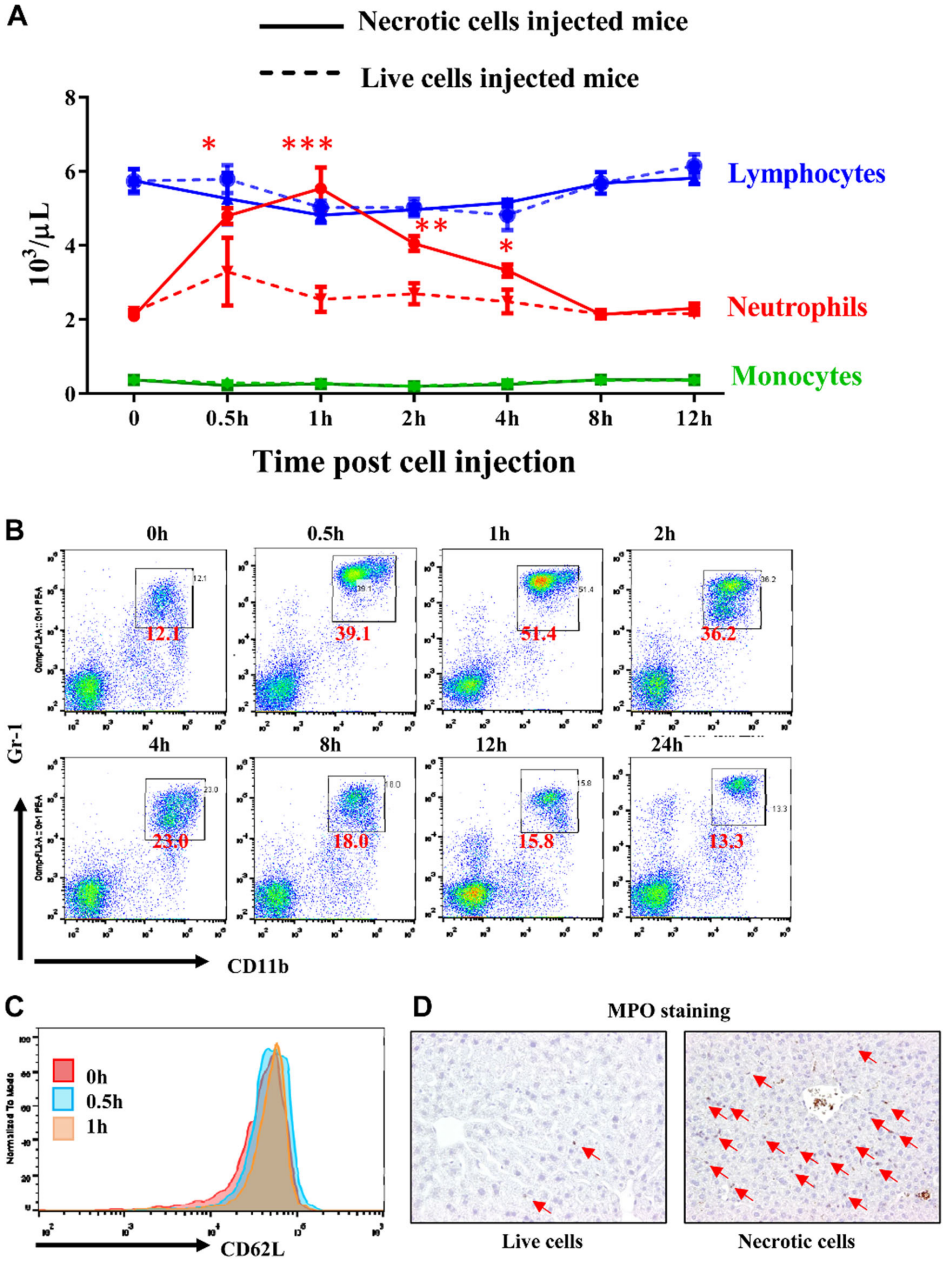
Necrotic cells induce rapid neutrophil mobilization. **a** Male B6 mice (6–10 weeks) were treated with necrotic or live HEK293 cells. Complete blood count were measured at different time points after necrotic cells (solid lines) or live cells (dashed lines) treatment. **b** Flow cytometry analysis of CD11b+Gr-1+ neutrophil percentage in PBMCs at different time points after necrotic cells treatment. **c** CD62L expression levels in CD11b+Gr-1+ neutrophil were compared at 0 h, 30 min, and 1 h after necrotic cells treatment. **d** Myeloperoxidase (MPO) staining in the liver from mice 1h after live or necrotic cells treatment. Arrows indicated MPO+ neutrophils. Magnification ×200. Data are expressed as mean ± SEM, *n* = 8 per group. *P < 0.05, **P < 0.05, ***P < 0.001

To investigate the acute inflammatory response of necrotic cells, live HEK293 cells, or necrotic HEK293 cells (after 3 freeze-thaw cycles) and injected into mice intravenously as described previously^14^. A complete blood count (CBC) was measured at different time points after injections. The blood neutrophil numbers significantly increased as early as 30 min after injections and sustained high levels until 4 h after injections (Fig.  1a, red solid line). Meanwhile blood monocyte and lymphocyte numbers stayed in normal range post necrotic cell treatment. In contrast, we did not see the significant changes in all three cell types when live cells were injected (Fig. 1a, dashed lines). The increase of neutrophils was further confirmed by flow cytometry analysis of peripheral blood mononuclear cells (PBMCs), the percentage of CD11b+Gr-1+ neutrophils increased from ~12 to ~40% within 30 min after necrotic cell treatment and peaked 1 h after treatment with >50% neutrophils in PBMCs (Fig. 1b). We did not observe a significant reduction of CD62L (a marker for neutrophil activation) expression on neutrophils with necrotic cell treatment, which indicated the neutrophils were not activated by necrotic cells (Fig. 1c). In addition, we found massive neutrophils infiltrated into organs such as liver when challenged by necrotic cells (Fig. 1d).

**Fig.2:**
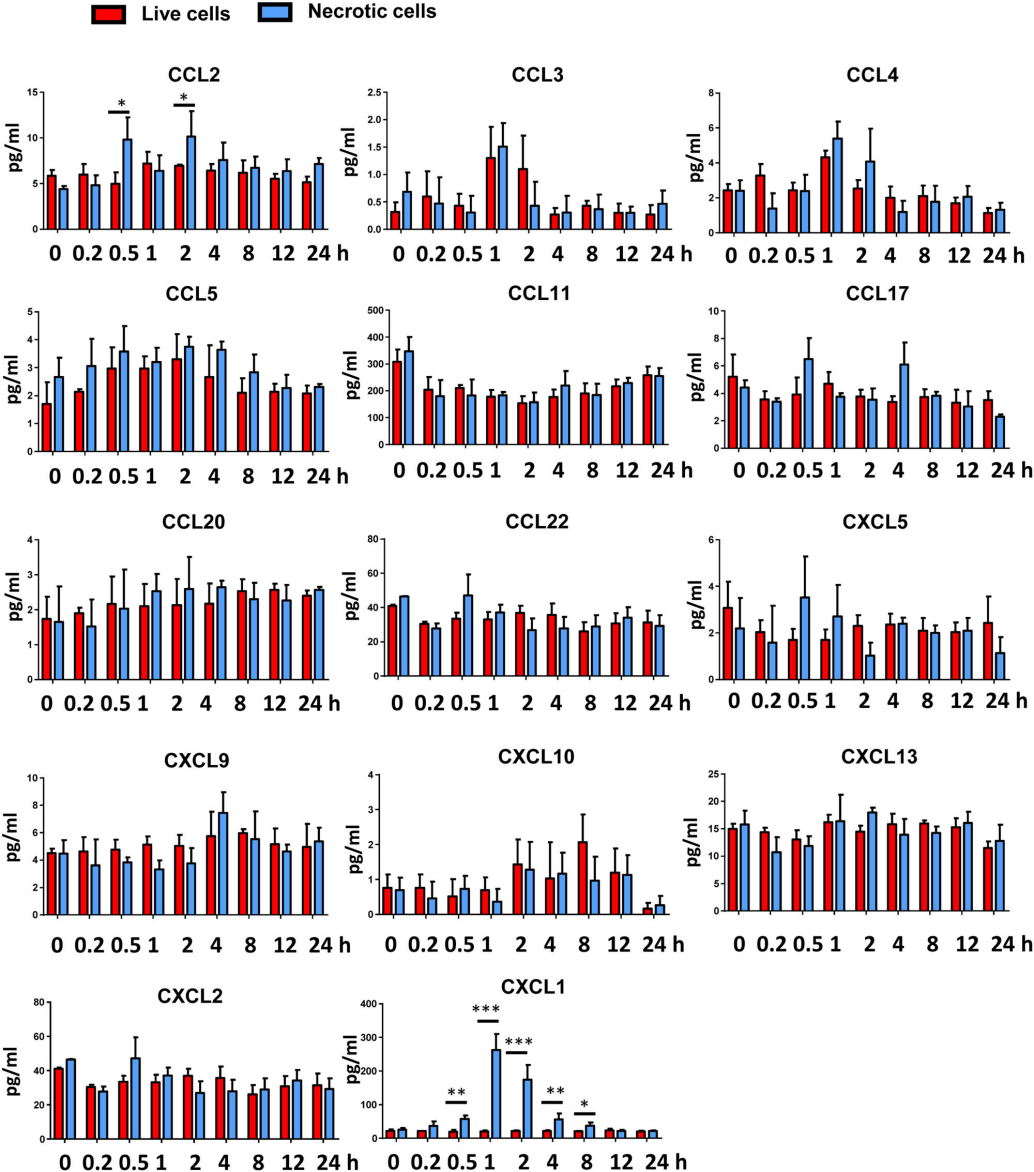
Necrotic cells induced rapid increase of CXCL1. B6 mice (6–10 weeks) were treated with necrotic or live HEK293 cells. Serum proinflammatory chemokines (CCL2, CCL3, CCL4, CCL5, CCL11, CCL17, CCL20, CCL22, CXCL1, CXCL2, CXCL5, CXCL9, CXCL10, and CXCL13) were measured by a beads-based assay or ELISA at different time points after treatment. Data are expressed as mean ± SEM, *n *= 6 per group. *P < 0.05, **P < 0.05, ***P < 0.001

We then asked which factor(s) was required for neutrophil mobilization in response to cell death in vivo. We employed a beads-based assay that can measure 13 proinflammatory chemokines (CCL2, CCL3, CCL4, CCL5, CCL11, CCL17, CCL20, CCL22, CXCL1, CXCL5, CXCL9, CXCL10, and CXCL13) simultaneously. We also checked another chemokine CXCL2, which was important in neutrophil chemotaxis^15^. As shown in Fig. 2, most of these chemokines remained in basal levels when treated with necrotic cells. Only the levels of CXCL1, which is considered as a critical and specific chemokine for promoting neutrophil migration, were markedly (~10 times higher compared with basal levels) elevated after necrotic cell treatment. The results were in agree with the observation that only neutrophils but not monocytes or lymphocytes were mobilized. As shown in Fig. 2, the elevation of mice serum CXCL1 levels was observed in response to necrotic cell challenge, whereas no significant change in serum CXCL1 levels when injected with live HEK293 cells. Consistent with neutrophil mobilization time course, the serum levels of CXCL1 also increased around half hour and returned to normal range 8 h after necrotic cell treatment. The peak levels of CXCL1 were found at 1 h after necrotic cell challenge.

**Fig.3:**
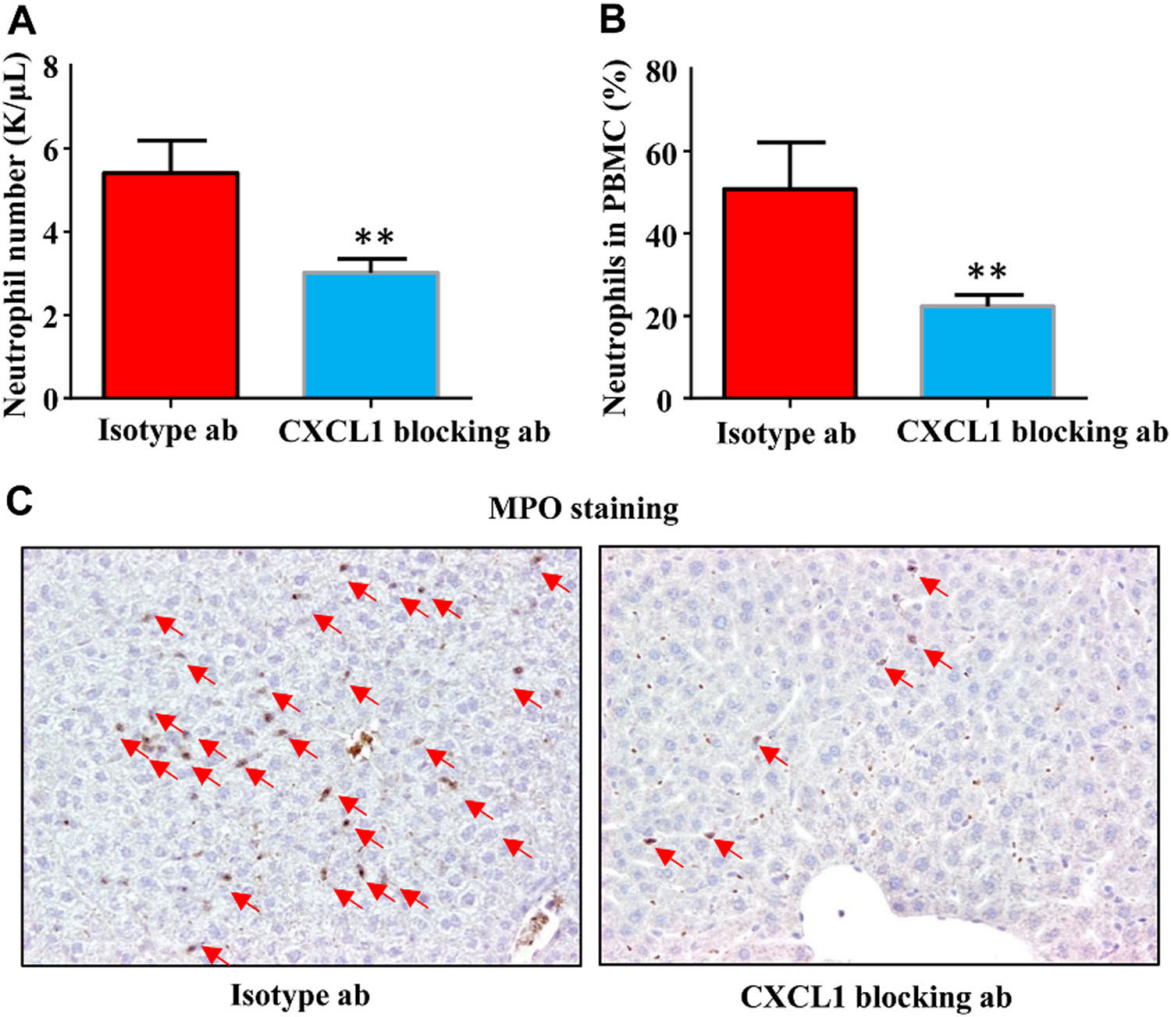
Necrotic cells induced neutrophil mobilization is CXCL1 dependent. Male B6 mice (6–10 weeks) were treated with necrotic or live HEK293 cells. Control isotype antibody or CXCL1 blocking antibody (100 μg per mouse) was injected 15 min before necrotic cells treatment, total blood neutrophil number (**a**) and neutrophil percentage (**b**) were determined 1 h after necrotic cells treatment. **c** Mice were pre-treated with control or CXCL1 blocking antibody as **a** and **b**, liver samples were obtained and stained with MPO antibody 1 h after necrotic cells treatment. Arrows indicated MPO+ neutrophils. Magnification ×200. Data are expressed as mean ± SEM, *n* = 6 per group. **P < 0.01, ***P < 0.001

To verify the role of CXCL1 in the neutrophil mobilization in response to necrotic cells, we treat the mice with CXCL1 blocking antibody prior to necrotic cell injections. As expected, neutralizing CXCL1 significantly blocked the increase of blood neutrophils as well as infiltrated liver neutrophils (Fig. 3a–c). These data suggested cell death-induced neutrophil mobilization was mediated by the elevation of serum CXCL1 levels.

### Liver is the major source of CXCL1 in response to cell death

**Fig.4:**
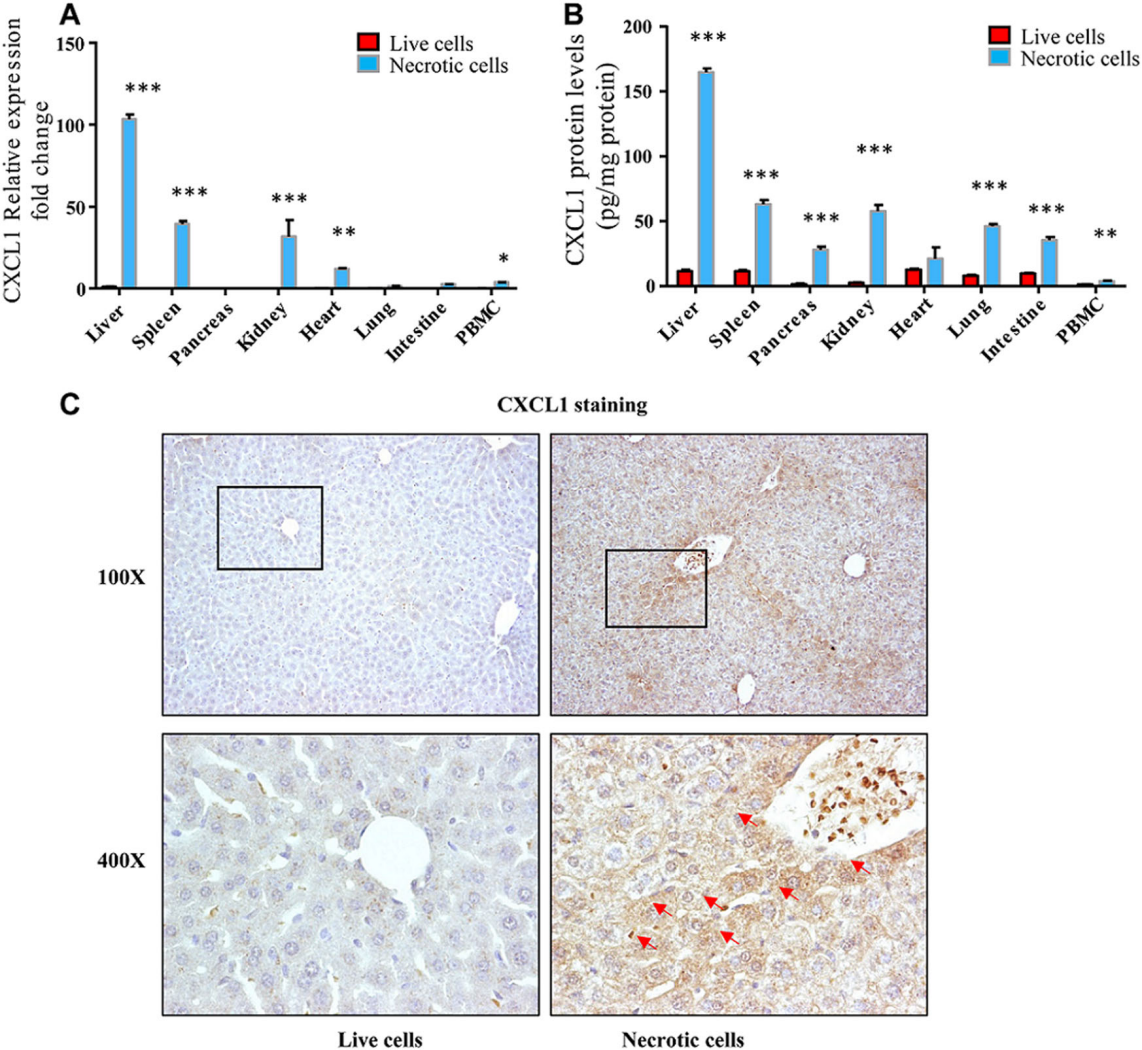
CXCL1 is mainly produced from hepatocytes in the liver. Male B6 mice (6–10 weeks) were treated with necrotic or live HEK293 cells. mRNA or tissue homogenates were obtained in various organs or PBMCs 1 h after necrotic or live cells treatment. CXCL1 mRNA levels (**a**) and CXCL1 protein levels (**b**) were measured by quantitative real-time PCR or ELISA. **c** Liver samples were obtained from the mice treated as **a** and **b** were stained with CXCL1 antibody. Arrows indicated CXCL1+ hepatocytes. Magnification ×100 and ×400. Data are expressed as mean ± SEM, *n *= 6 per group. *P < 0.05, **P < 0.01, ***P < 0.001

We next asked what was the main source of CXCL1 in response to necrotic cell treatment. CXCL1 mRNA levels were determine by quantitative RT-PCR in several major organs and PBMCs. As shown in Fig. 4a, CXCL1 mRNA was significantly increased in spleen, kidney, heart, and liver 1 h after necrotic cell treatment. Surprisingly, in the liver, CXCL1 mRNA levels increased more than 100-folds compared with live HEK293 cell treatment. Also, the mRNA levels of CXCL1 in the liver were more than two times higher than in spleen, kidney and 5 to 20 times higher than in other organs. In agree with mRNA results, we found the highest CXCL1 protein levels in the liver compared with other organs and PBMCs (at least more than two times higher than other organs and PBMCs as shown in Fig. 4b). Of note, liver is the largest solid organ in the body, so our data showed that the liver is the main source of increase CXCL1 in response to necrotic cell challenge. To further clarify which cell type produce CXCL1, we performed CXCL1 immunohistochemical staining in the liver 1 h after challenge. As shown in Fig. 4c, most CXCL1 positive cells were hepatocytes by morphology. Interestingly, CXCL1 positive hepatocytes preferentially located around portal vein and central vein area. Taken together, the liver contributes to the major part of CXCL1 production in necrotic cell challenge.

### Kupffer cells-derived TNF-α is required for CXCL1 production

**Fig.5:**
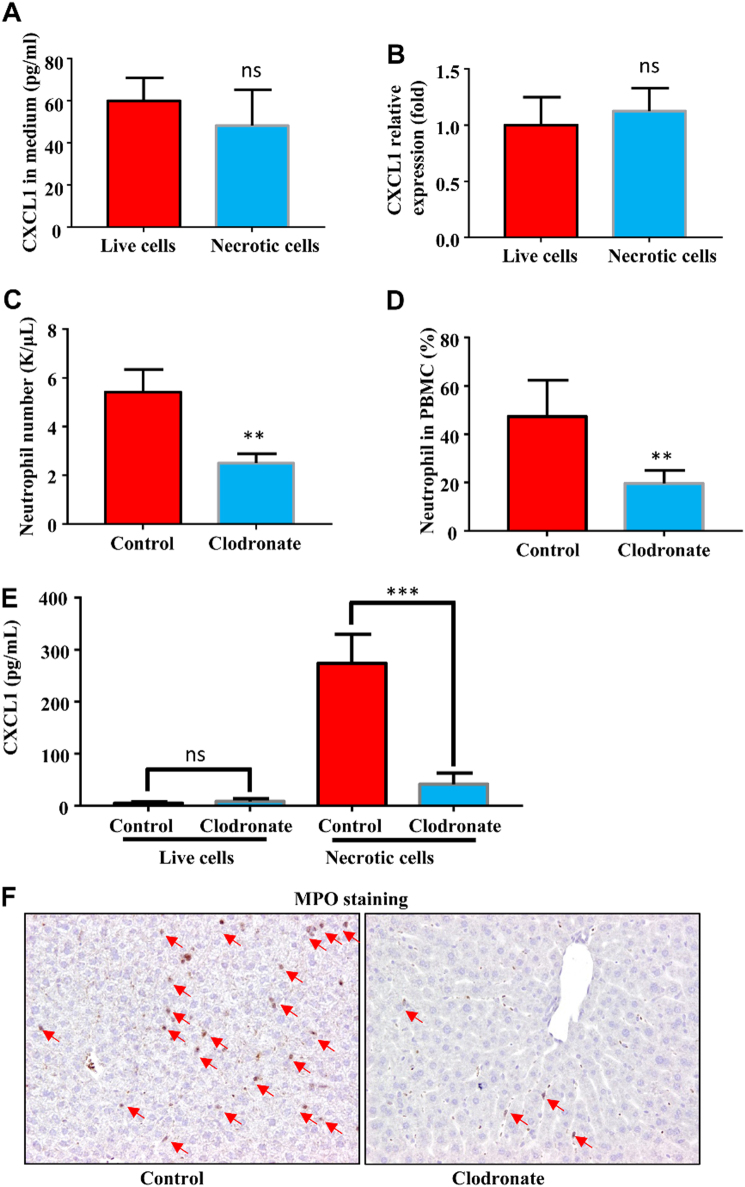
Ablation of Kupffer cells abolishes the CXCL1 production and neutrophil mobilization. Hepatocytes were isolated from male B6 mice (6–10 weeks) were treated with necrotic or live HEK293 cells. CXCL1 mRNA levels of hepatocytes (**a**) and CXCL1 protein levels in the culture medium (**b**) were measured by quantitative real-time PCR or ELISA. Male B6 mice (6–10 weeks) were treated with clodronate liposomes or control liposomes, 24 h later all mice received necrotic cells or live cells. **c** Serum CXCL1 levels were measured 1 h after cell treatment. Total blood neutrophil number (**d**) and neutrophil percentage (**e**) were determined 1 h after necrotic cells treatment. **f** Liver samples were obtained and stained with MPO antibody 1 h after necrotic cells treatment. Arrows indicated MPO+ neutrophils. Magnification ×200. Data are expressed as mean ± SEM, *n *= 6 per group. **P < 0.01, ***P < 0.001

We next asked how hepatocytes response to the necrotic cells to produce CXCL1. To test the possibility of the hepatocyte can directly produce CXCL1 with necrotic cells challenge, we freshly isolated hepatocytes and co-cultured with live and necrotic HEK293 cells. As shown in Fig. 5a, the CXCL1 protein levels in the medium from the co-culture experiments were similar by comparing the hepatocytes/live HEK293 co-culture to the hepatocytes/necrotic co-culture, and the CXCL1 mRNA levels in hepatocyte treated with liver and necrotic cells were detected at the similar expression level with no significant difference (Fig. 5b). These data suggested that necrotic cells could not directly stimulate the hepatocytes to produce CXCL1 in vitro, there must be other intermediate pathways that have critical roles in between.

As previously reported, the liver-resident macrophages, Kupffer cells had important role in clearance of bacterial and bacterial products by phagocytosis and cytokine production^16^. We hypothesized that Kupffer cells might be critical in sensing necrotic cells and promoting CXCL1 production in hepatocytes. To test the hypothesis, we ablated Kupffer cells by treating mice with clodronate liposome. As shown in Fig. S1, the F4/80-positive Kupffer cells were profoundly depleted upon clodronate liposome treatment compared with control liposome treatment, with almost no F4/80-positive cells detected in the liver histologically. We then treated the Kupffer cell-ablated mice or control liposome-treated mice with necrotic cells, significant decrease neutrophil mobilization was observed in the Kupffer cell-ablated mice treated with necrotic cells, evidenced by significantly lower both number and percentage of neutrophils in Kupffer cell-ablated mice compared with control liposome-treated mice (Fig. 5c, d). Blood serum CXCL1 levels were measured, as shown in Fig. 5e, the CXCL1 levels in Kupffer cell-ablated mice treated with necrotic cells is significantly lower than in the control liposome-treated mice challenged with necrotic cells. The reduction of liver neutrophils was also observed in Kupffer cell-ablated mice post necrotic cells treatment (Fig. 5f).

**Fig.6:**
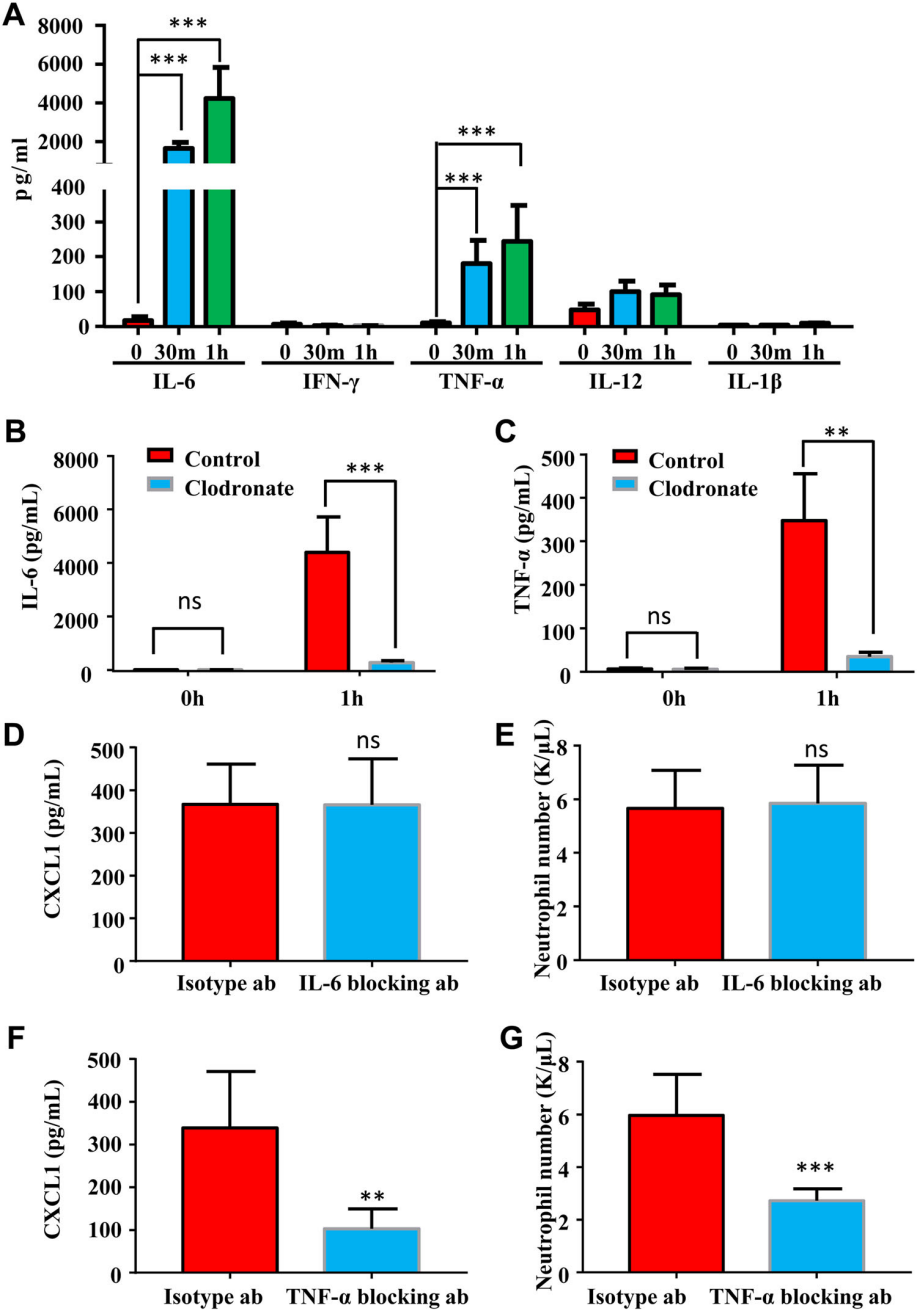
TNF-α but not IL-6 mediates the production of CXCL1 in hepatocytes. Male B6 mice (6~10 weeks) were treated with necrotic cells. **a** Serum levels of proinflammatory cytokines such as IL-6, IFN-γ, TNF-α, IL-12, IL-1β were measured 0, 30 min, and 1 h after necrotic cells treatment. **b**, **c** Male B6 mice (6–10 weeks) were pre-treated with clodronate liposomes or control liposomes, 24 h later mice received necrotic cells. Serum IL-6 and TNF-α levels were measured 1 h after cell treatment. **d**–**g** Control isotype antibody or IL-6, TNF-α blocking antibody (100 ng per mouse) was injected 15 min before necrotic cells treatment, CXCL1 concentration (**d**, **f**) and neutrophil number (**e**, **g**) were determined 1 h after necrotic cells treatment. Data are expressed as mean ± SEM, *n *= 6 per group. **P < 0.01, ***P < 0.001

Kupffer cells can produce several proinflammatory cytokines in response to bacterial products^16^, these cytokines, including IL-6, IL-1β, IFN-γ, IL-12, and TNF-α, are very important in promoting the activation of immune cells and acute-phase protein production in hepatocyte^17,18^. Here we also checked how the production of these cytokines were regulated by necrotic cell challenge and which cytokine(s) is required for CXCL1 production by hepatocytes within 1 h after challenge. We measured serum levels of IL-6, IL-1β, IFN-γ, and TNF-α in early time points (0 h, 0.5 h, and 1h) with necrotic cell treatments. As shown in Fig. 6a, we observed significant elevation of IL-6 and TNF-α but not IL-1β, IFN-γ, and IL-12 at 0.5 h, and 1h after necrotic cell treatment. Ablation of Kupffer cells almost completely abolished the elevation of IL-6 and TNF-α (Fig. 6b, c). We then focused on IL-6 and TNF-α to clarify whether they were involved in hepatocyte CXCL1 production. IL-6 and TNF-α blocking antibodies or isotype antibodies were administrated 30 min prior to necrotic cell treatment. We found that neutralizing IL-6 did not affect the necrotic cell-induced neutrophil mobilization as well as CXCL1 production (Fig. 6d, e). In contrast, blocking antibody for TNF-α significantly reduced the elevation of both blood neutrophil number and serum CXCL1 levels with necrotic cell challenge (Fig. 6f, g). These data supported that Kupffer cell-derived TNF-α was critical in hepatocyte CXCL1 production after necrotic cell challenge.

### CXCL1 production is NF-κB dependent

**Fig.7:**
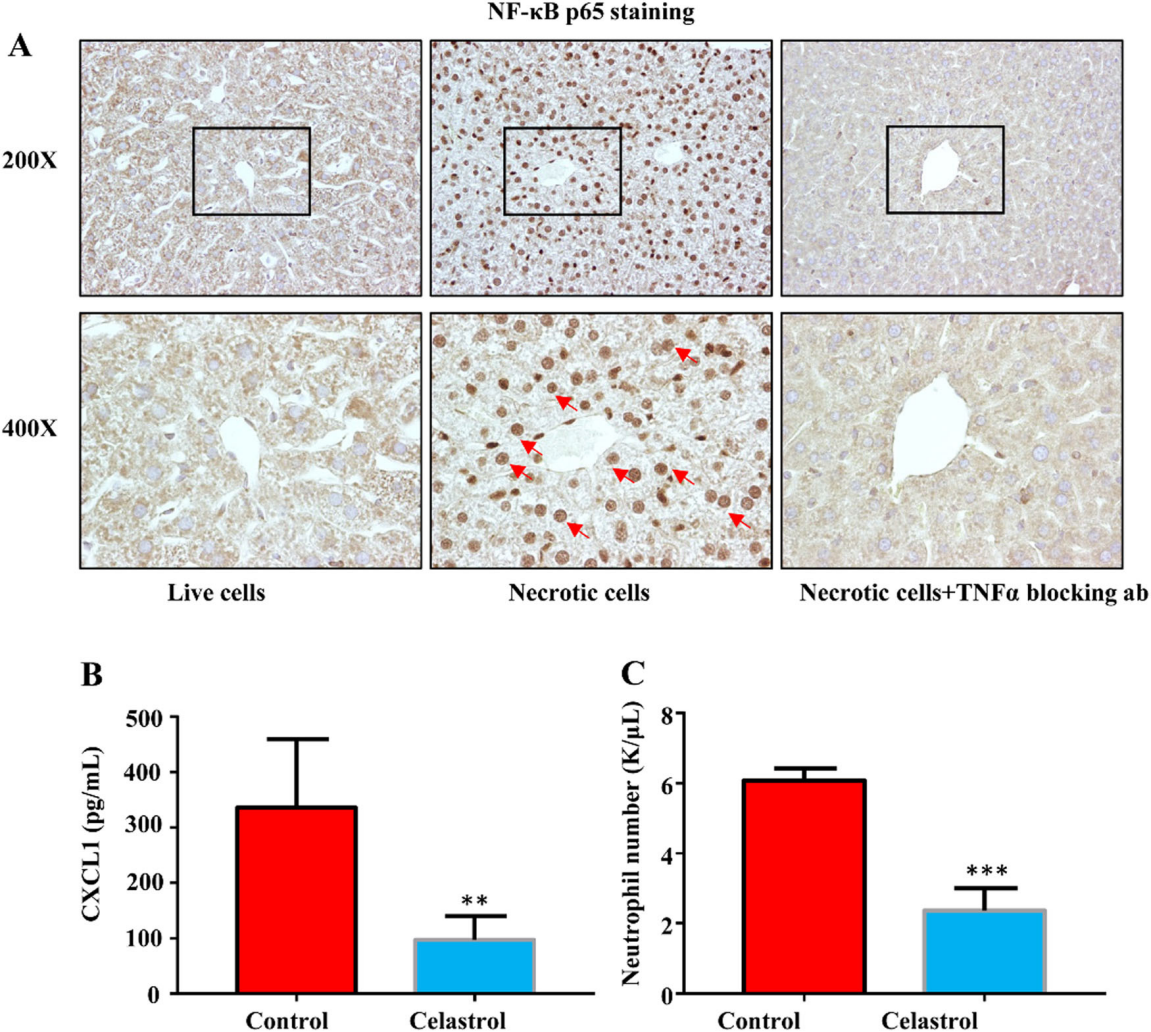
NF-κB activation is required for hepatocyte CXCL1 production. Male B6 mice (6–10 weeks) were treated with live cells, necrotic cells or necrotic cells with TNF-α blocking antibody pretreatment. **a** Liver samples were obtained and stained with NF-κB P65 antibody, arrows indicated NF-κB P65 nuclear staining in hepatocytes. Magnification ×200 and ×400. Control or NF-κB inhibitor Celastrol (100 µg/kg) was injected 3 h before necrotic cells treatment, total blood neutrophil number (**b**), and neutrophil percentage (**c**) were determined 1 h after necrotic cells treatment. Data are expressed as mean ± SEM, *n* = 6 per group. **P < 0.01, ***P < 0.001

Previous studies suggested that CXCL1 production required NF-κB activation^19–21^. Also, TNF-α was a well-established upstream activator of NF-κB signaling^22,23^. So, we asked whether TNF-α/NF-κB signaling was involved in necrotic cell-induced CXCL1 production. We perform immunohistochemical staining of NF-κB P65 on the liver. As shown in Fig. 7a, we observed P65 located in the cytoplasm of hepatocyte in control mice, however, necrotic cell challenge led the exclusively nuclear localization of NF-κB P65, which was considered as a marker for NF-κB activation^24^. In contrast, the neutralization of TNF-α by antibody significantly blocked the nuclear localization of NF-κB P65, which confirmed the role of Kupffer cell-derived TNF-α in hepatocyte NF-κB activation.

To further verify the role of NF-κB in hepatocyte CXCL1 production, we pre-treated mice with Celastrol, an inhibitor of TNF-α-induced NF-κB activation,^25^ prior to necrotic cell challenge. Celastrol significantly blocked the elevation of serum CXCL1 levels as well as blood neutrophil number and percentage (Fig. 7b, c). These data supported that necrotic cell-induced CXCL1 production was TNF-α/NF-κB dependent.

### Discussion

**Fig.8:**
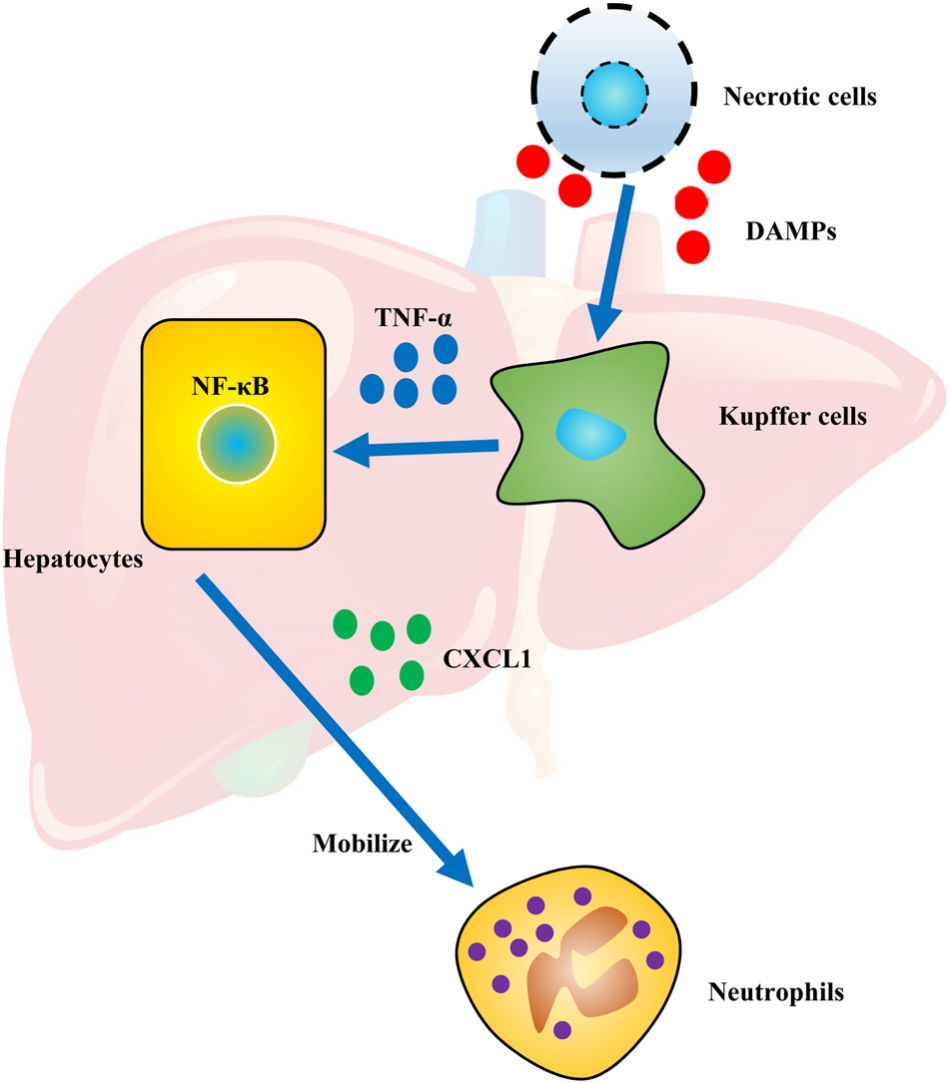
**A summarized figure depicting proposed mechanisms for how necrotic cell-derived DAMPs mobilize neutrophils**

In current study, we showed that necrotic cell could trigger neutrophil mobilization in CXCL1-dependent manner. Our results revealed that the liver was the major source of CXCL1 production upon necrotic cell challenge, the presence and interaction between Kupffer cells and hepatocytes was critical for CXCL1 production, the Kupffer cells could elevate the TNF-α production to activate NF-κB in hepatocytes and promote hepatocytes to produce CXCL1 for neutrophil mobilization upon necrotic cell stimulation (summarized in Fig. 8).

As the largest solid organ in the body, liver has critical roles in energy metabolism, detoxification, glycogen storage, hormone production, and protein synthesis. Recently, the liver was considered as an immunological organ with predominate innate immunity^26,27^. Innate immune cells, including Kupffer cells, NK cells, NKT cells are enriched in the liver. Upon infection, these innate immune cells directly respond to PAMPs including bacterial and bacterial products from intestine by phagocytosis and cytokine production^26^. In addition to immune cells, hepatocytes in the liver can also produce large amount of acute-phase proteins (APPs) with PAMP stimulation^28^. The production of APPs requires STAT3 and NF-κB signaling activation in hepatocytes^17^. These APPs have important roles in killing pathogens directly or supporting efficient immune response^29–31^. As discussed above, how the liver contributes to fight against the invading pathogens has been extensively studied, however, how the liver responses to DAMP remains elusive. Although it was reported that chemokines were elevated with necrotic cell treatment^6^. The main origin of these chemokines is not clear. In this study, we checked multiple proinflammatory chemokines production, including neutrophil chemotaxis-related chemokines CXCL1, CXCL2, and CXCL5, upon necrotic cell treatment and found that CXCL1 was the most significantly elevated chemokine with necrotic cell challenge (Fig. 2) and confirmed that CXCL1 was responsible for neutrophil mobilization (Fig. 3). In our model, we did see a slight increase of CCL2 in 0.5 and 2 h after necrotic cell challenge. However, the absolute concentration of CCL2 was still very low (~10 pg/ml) compared with infection models (500–3000 pg/ml)^32^. This was consistent with the observation that monocytes and lymphocytes percentages in PBMCs were not changed in our model. Although, we found neutrophil-specific chemokine CXCL1 mRNA and protein levels increased in several organs and PBMC with necrotic cell challenge, hepatocytes in the liver were the major source of CXCL1 (Fig. 4). Moreover, our results indicated that Kupffer cell-derived TNF-α and TNF-α downstream NF-κB signaling were critical in CXCL1 production from hepatocytes. To our surprise, although the serum levels of IL-6 increased by 200 times upon necrotic cell challenge, we did not see impaired of CXCL1 production as well as neutrophil mobilization when IL-6 was neutralized by antibody pretreatment (Fig. 6a, d, e). This phenomenon may be explained by the lack of STAT3 binding sites in the promoter region of CXCL1 while NF-κB binding site was identified in that region^20,23,33^. As another well-known NF-κB activator, IL-1β is considered as an important mediator in PAMPs induced inflammation^34^. Here we also checked the serum levels of IL-1β when challenged with necrotic cells, however, no significant change was observed in early response to necrotic cells (Fig. 6b), suggesting that IL-1β is not involved in the neutrophil mobilization during early response to DAMPs challenge.

Hepatocyte is the major cell type of APPs synthesis in response to cytokines. Many proinflammatory cytokine receptors such as IL-6 receptor, TNF-α receptor, IL-1β receptor, and IFN-γ receptor are expressed in hepatocytes. Although several reports showed that hepatocytes can produce these inflammatory cytokines under certain conditions^35,36^, innate immune cells, especially Kupffer cells in the liver are believed to the main source of proinflammatory cytokines during acute infections^16,37^. We tried to verify whether similar things happen to DAMPs. We showed that the hepatocytes cannot produce CXCL1 when treated with necrotic cells directly (Fig. 5a, b). Of note, Kupffer cells are the major phagocytes to clear dead cells^16^. So, we speculate Kupffer cells are the first responders to produce inflammatory cytokines upon necrotic cell challenge. Indeed, we observed rapid inflammatory cytokines (such as IL-6 and TNF-α) increase with necrotic cell challenge and the increase were abolished when Kupffer cells were ablated (Fig. 6a). As the Kupffer cells are closely associated with hepatocytes, a higher liver local concentration of proinflammatory cytokines will facilitate hepatocytes to produce CXCL1 promptly and efficiently.

Taken together, our findings provide a good example of orchestration between liver-resident Kupffer cells and hepatocytes in response to DAMP challenge (Fig. 8). The liver has key role in sensing DAMPs, producing CXCL1 to mobilize neutrophils and finally clearing dead cells.

## Electronic supplementary material

Fig supplement

Supplementary Figure Legend

## References

[1] Tang D, Kang R, Coyne CB, Zeh HJ, Lotze MT (2012). PAMPs and DAMPs: signal 0s that spur autophagy and immunity. Immunol. Rev..

[2] Venereau E, Ceriotti C, Bianchi ME (2015). DAMPs from cell death to new life. Front. Immunol..

[3] Bianchi ME (2007). DAMPs, PAMPs and alarmins: all we need to know about danger. J. Leukoc. Biol..

[4] Sachet M, Liang YY, Oehler R (2017). The immune response to secondary necrotic cells. Apoptosis.

[5] Zitvogel L, Kepp O, Kroemer G (2010). Decoding cell death signals in inflammation and immunity. Cell.

[6] Tanimoto N (2007). Involvement of KC, MIP-2, and MCP-1 in leukocyte infiltration following injection of necrotic cells into the peritoneal cavity. Biochem. Biophys. Res. Commun..

[7] Kobayashi Y (2008). The role of chemokines in neutrophil biology. Front. Biosci..

[8] Furze RC, Rankin SM (2008). Neutrophil mobilization and clearance in the bone marrow. Immunology.

[9] Bleul CC, Fuhlbrigge RC, Casasnovas JM, Aiuti A, Springer TA (1996). A highly efficacious lymphocyte chemoattractant, stromal cell-derived factor 1 (SDF-1). J. Exp. Med..

[10] Martin C (2003). Chemokines acting via CXCR2 and CXCR4 control the release of neutrophils from the bone marrow and their return following senescence. Immunity.

[11] Alvarenga DM, Mattos MS, Araujo AM, Antunes MM, Menezes GB (2018). Neutrophil biology within hepatic environment. Cell Tissue Res..

[12] Papayannopoulos, V. Neutrophil extracellular traps in immunity and disease. *Nat. Rev. Immunol*. **18**, 134–147 (2017). 10.1038/nri.2017.105 28990587

[13] Zhang Q (2010). Circulating mitochondrial DAMPs cause inflammatory responses to injury. Nature.

[14] Neumann K (2014). Clec12a is an inhibitory receptor for uric acid crystals that regulates inflammation in response to cell death. Immunity.

[15] Kolaczkowska E, Kubes P (2013). Neutrophil recruitment and function in health and inflammation. Nat. Rev. Immunol..

[16] Bilzer M, Roggel F, Gerbes AL (2006). Role of Kupffer cells in host defense and liver disease. Liver. Int..

[17] Quinton LJ (2012). Hepatocyte-specific mutation of both NF-kappa B RelA and STAT3 abrogates the acute phase response in mice. J. Clin. Invest..

[18] Schumann J (2000). Importance of Kupffer cells for T-cell-dependent liver injury in mice. Am. J. Pathol..

[19] Burke SJ (2014). NF-kappa B and STAT1 control CXCL1 and CXCL2 gene transcription. Am. J. Physiol. Endocrinol. Metab..

[20] Wood LD, Richmond A (1995). Constitutive and cytokine-induced expression of the melanoma growth stimulatory activity/GRO alpha gene requires both NF-kappa B and novel constitutive factors. J. Biol. Chem..

[21] Wood LD, Farmer AA, Richmond A (1995). HMGI(Y) and Sp1 in addition to NF-kappa B regulate transcription of the MGSA/GRO alpha gene. Nucleic Acids Res..

[22] Hsu H, Xiong J, Goeddel DV (1995). The TNF receptor 1-associated protein TRADD signals cell death and NF-kappa B activation. Cell.

[23] Liu ZG, Hsu H, Goeddel DV, Karin M (1996). Dissection of TNF receptor 1 effector functions: JNK activation is not linked to apoptosis while NF-kappa B activation prevents cell death. Cell.

[24] Beg AA (1992). I kappa B interacts with the nuclear localization sequences of the subunits of NF-kappa B: a mechanism for cytoplasmic retention. Genes Dev..

[25] Sethi G, Ahn KS, Pandey MK, Aggarwal BB (2007). Celastrol, a novel triterpene, potentiates TNF-induced apoptosis and suppresses invasion of tumor cells by inhibiting NF-kappa B-regulated gene products and TAK1-mediated NF-kappa B activation. Blood.

[26] Gao B, Jeong WI, Tian Z (2008). Liver: an organ with predominant innate immunity. Hepatology.

[27] Racanelli V, Rehermann B (2006). The liver as an immunological organ. Hepatology.

[28] Zhou Z, Xu MJ, Gao B (2016). Hepatocytes: a key cell type for innate immunity. Cell Mol. Immunol..

[29] Inatsu A (2009). Novel mechanism of C-reactive protein for enhancing mouse liver innate immunity. Hepatology.

[30] Shah C, Hari-Dass R, Raynes JG (2006). Serum amyloid A is an innate immune opsonin for Gram-negative bacteria. Blood.

[31] Xu MJ (2015). Liver is the major source of elevated serum lipocalin-2 levels after bacterial infection or partial hepatectomy: a critical role for IL-6/STAT3. Hepatology.

[32] Bardina SV (2015). Differential roles of chemokines CCL2 and CCL7 in monocytosis and leukocyte migration during West Nile virus infection. J. Immunol..

[33] Anisowicz A, Messineo M, Lee SW, Sager R (1991). An NF-kappa B-like transcription factor mediates IL-1/TNF-alpha induction of gro in human fibroblasts. J. Immunol..

[34] Miura K (2010). Toll-like receptor 9 promotes steatohepatitis by induction of interleukin-1beta in mice. Gastroenterology.

[35] Norris CA (2014). Synthesis of IL-6 by hepatocytes is a normal response to common hepatic stimuli. PLoS ONE.

[36] Takano M (2012). Hepatocytes produce tumor necrosis factor-alpha and interleukin-6 in response to *Porphyromonas gingivalis*. J. Periodontal. Res..

[37] Boltjes A, Movita D, Boonstra A, Woltman AM (2014). The role of Kupffer cells in hepatitis B and hepatitis C virus infections. J. Hepatol..

