# Supplementary figures and images for: Kupffer cell-derived TNF-α promotes hepatocytes to produce CXCL1 and mobilize neutrophils in response to necrotic cells
Source: Cell Death Dis. 2018 Feb 23;9(3):323. doi: 10.1038/s41419-018-0377-4 (PMC5833701; doi:10.1038/s41419-018-0377-4)

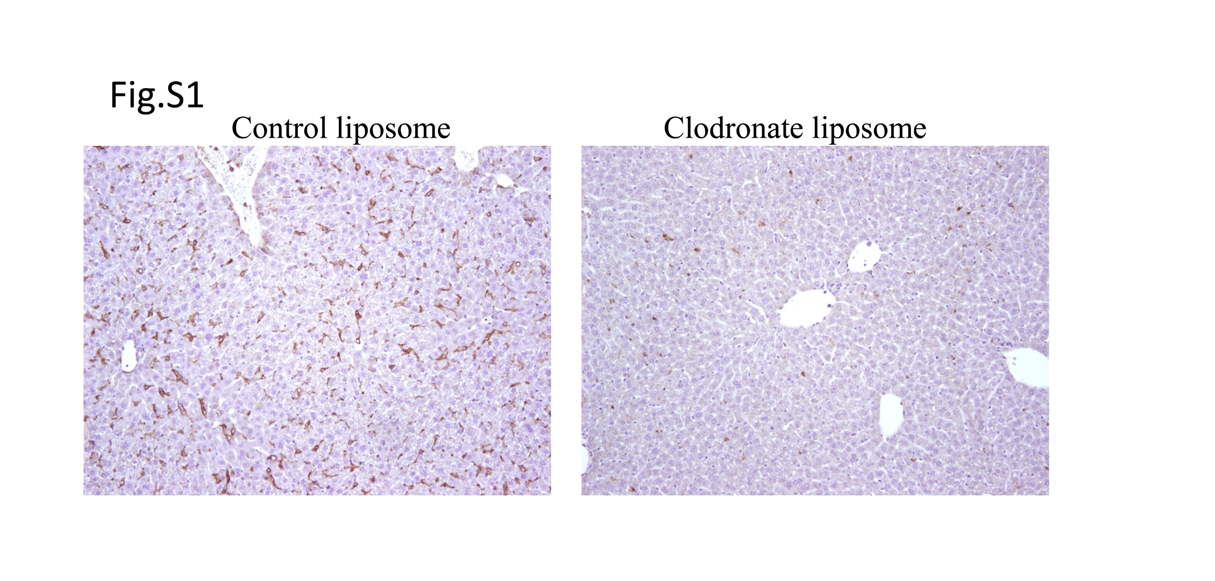

Supplement: Supplementary file 1 — Fig supplement [file 41419_2018_377_MOESM1_ESM.tif]
