# Supplementary material for: Kupffer cell-derived TNF-α promotes hepatocytes to produce CXCL1 and mobilize neutrophils in response to necrotic cells
Source: Cell Death Dis. 2018 Feb 23;9(3):323. doi: 10.1038/s41419-018-0377-4 (PMC5833701; doi:10.1038/s41419-018-0377-4)
Supplement: Supplementary file 2 — Supplementary Figure Legend [file 41419_2018_377_MOESM2_ESM.docx]

**Supplementary Figure Legend**

**Fig. S1 Efficiency of F4/80+ Kupffer cell ablation by clodronate liposome treatment.** Mice were treated with control liposome or clodronate liposome, 24 hours later, liver samples were obtained and stained with F4/80 antibody. Magnification 100X
